# In Vitro Evaluation of the Antimicrobial Properties of Nanoparticles as New Agents Used in Teat Sealants for Mastitis Prevention in Dry Cows

**DOI:** 10.3390/biomedicines11082291

**Published:** 2023-08-17

**Authors:** Daniel Radzikowski, Aleksandra Kalińska, Magdalena Kot, Sławomir Jaworski, Mateusz Wierzbicki, Marcin Gołębiewski

**Affiliations:** 1 Animal Breeding Department, Warsaw University of Life Sciences, 02-786 Warszawa, Poland; aleksandra_kalinska@sggw.edu.pl (A.K.); magdalena_kot@sggw.edu.pl (M.K.); 2 Department of Nanobiotechnology, Warsaw University of Life Sciences, 02-786 Warszawa, Polandmateusz_wierzbicki@sggw.edu.pl (M.W.)

**Keywords:** silver, copper, gold, nanoparticles, mastitis, dry cows

## Abstract

Mastitis prevention and treatment in dry cows are complex issues with limited solutions. The most common is intramammary antibiotic treatment. However, the effectiveness of this treatment varies among countries and even within herds in the same region. Therefore, it is necessary to develop new strategies for dry cow therapy. Metal nanoparticles (NPs), which have strong biocidal properties for treating diseases caused by bacteria, fungi, and algae, are increasingly used to reduce antibiotic use. In this study, AuNPs, CuNPs, AgNPs, PtNPs, NP-FeCs, and their triple complexes were used at different concentrations to evaluate their practical use in treating cows during their dry period. The nanoparticles were in hydrocolloid form and were added separately to form a mixture with beeswax, a mixture with oil, or a mixture based on vegetable glycerin and propylene glycol. The NPs’ concentrations were 0.5, 1, 2, and 5 ppm. Gram-positive and Gram-negative bacteria, and fungi isolated from cows diagnosed with mastitis were used to determine pathogen viability. The results indicated that AuNPs, CuNPs, AgNPs, and their complexes show biocidal properties against mastitis pathogens. AgNPs at 5 ppm had the strongest biocidal properties and reduced *Streptococcus agalactiae*’s survival rate by 50%; however, the nanoparticle complexes showed poor synergism. The strongest biocidal properties of NPs in wax and in glycerin mixed with glycol were shown against *Escherichia coli*. Additionally, low nanoparticle concentrations showed no cytotoxicity for BME-UV1 bovine cells, suggesting that these mixtures might be used for further in vivo testing.

## 1. Introduction

In dairy cattle herds, a large percentage of cows are affected by mastitis—inflammation of the mammary gland. The consequences of the occurrence of this disease are a decrease in animal welfare and a loss of animal performance due to decreased milk production and the necessity for veterinary treatment. Mastitis is mainly caused by bacteria [1], fungi, or algae [2]. To prevent mastitis, an appropriate hygiene standard (hygienic surroundings and disinfection of milking equipment) is crucial; however, due to the conditions in the barn, this is challenging to accomplish. These pathogens may originate from various sources, for instance, from the environment in which the animal is living [3]. Infection can occur through pathogens penetrating the teat canal and then entering the mammary gland, where inflammation may occur. The reservoir for these pathogens may be found not only in the surrounding environment but also on contaminated tools, other animals, or staff who do not follow hygiene procedures [1]. Pathogens that cause mastitis can be divided into environmental and infectious groups. Environmental pathogens include *Escherichia coli*, *Klebsiella* spp., and *Candida* spp., while the infectious group includes *Staphylococcus aureus*, *Streptococcus agalactiae*, *Streptococcus dysgalactiae*, *Streptococcus uberis*, and *Mycoplasma bovis*. High temperatures and humidity promote the development of various fungi that can induce mastitis, including *Geotrichum* spp., *Trichosporon* spp., *Rhodotorula* spp., and *Torulopsis* spp. However, the *Candida* genus’s yeasts are deemed to be the prevailing mycotic flora [4]. There are also known cases of mastitis induced by algae from the genus *Prototheca* [2].

The most common treatment for mastitis is antibiotic therapy; however, the long-term overuse and application of antibiotics in animal production as a form of prophylaxis has led to the development of resistant bacteria, further leading to the development of antibiotic resistance [5], which poses a threat to animal [6] and human [7] health. Herd management during the dry period, which is the period with no milk production, is crucial. At the same time, the dry period forms the transitional period between the last lactation, and calving and the next lactation; therefore, in order to prevent a recurrence of the disease, it is necessary to implement treatment to cure the inflammation and also to focus on preventing the occurrence of mastitis [8]. Due to the growing problem of antibiotic resistance, it is essential to look for alternatives that can be used in the treatment and prophylaxis of mastitis. The latest and most innovative solution for eliminating bacterial growth is the use of nanoparticles (NPs). These particles effectively damage the cell membranes of bacteria and the biofilms they are able to form [9], especially in the case of drug-resistant bacteria, such as *Staphylococcus aureus*, *Staphylococcus epidermidis*, and *Escherichia coli* [10].

The aim of this study was to conduct an in vitro analysis to determine the effect of nanoparticle (NP) hydrocolloids of AuNPs, CuNPs, AgNPs, PtNPs, iron nanoparticles with a surface carbon layer (NP-FeCs), and their triple complexes on the survival of the most common mastitis pathogens. Homogenous NPs were tested in various forms: (1) hydrocolloid, (2) mixed with beeswax, (3) mixed with olive oil, and (4) in a mixture based on vegetable glycerin and propylene glycol. The mentioned mixtures were used against the pathogens *Staphylococcus aureus*, *Escherichia coli*, *Streptococcus agalactiae*, *Streptococcus uberis*, *Enterococcus faecalis*, *Enterobacter cloacae*, *Pseudomonas aeruginosa*, and *Candida albicans*. The effect of the NPs in hydrocolloid and mixture forms on the viability of BME-UV1 epithelial mammary cells was also determined.

## 2. Materials and Methods

### 2.1. Preparation of Homogenous Metal NPs and Their Complexes Using the Self-Organization Phenomenon

Solutions of NP complexes were prepared by mixing AgNP, CuNP, and AuNP hydrocolloids at a concentration of 50 mg/L (Nano-Tech, Warsaw, Poland) and NP-FeC hydrocolloids at a concentration of 50 mg/L (PlasmaChem, Berlin, Germany) at a 1:1 ratio. All the prepared NP solutions were incubated for 24 h at a temperature of 24 °C and then used for further analysis. Homogenous hydrocolloids were stored at room temperature. The NPs from Nano-Tech and the NP-FeCs from PlasmaChem were synthesized using physical methods. According to the first manufacturer, the NPs were produced using an innovative patented method for physically obtaining non-ionic precious and semi-precious metal NPs. The process is based on a method of physically obtaining NPs through the laboratory disintegration of pure metals into particles a few to several nanometers in size.

A quality certificate for each batch of NPs (e.g., concentration in ppm, pH, shelf life, and batch number) was delivered by the manufacturer as confirmation that the NPs were of the necessary quality to conduct studies. The stability of the AgNPs, CuNPs, and AuNPs was also confirmed by the manufacturer.

### 2.2. Physicochemical Properties of Metal NPs

The zeta potential and the distribution of the AuNPs’ and NP-FeCs’ sizes were determined, as these NPs had not been included in previous research; a Zetasizer Nano ZS (ZEN3500; Malvern Instruments, Malvern, UK) was used for this purpose. The zeta potential, mobility, and conductivity of the AuNPs and NP-FeCs at concentrations of 50 mg/L were also obtained.

**Figure 1:**
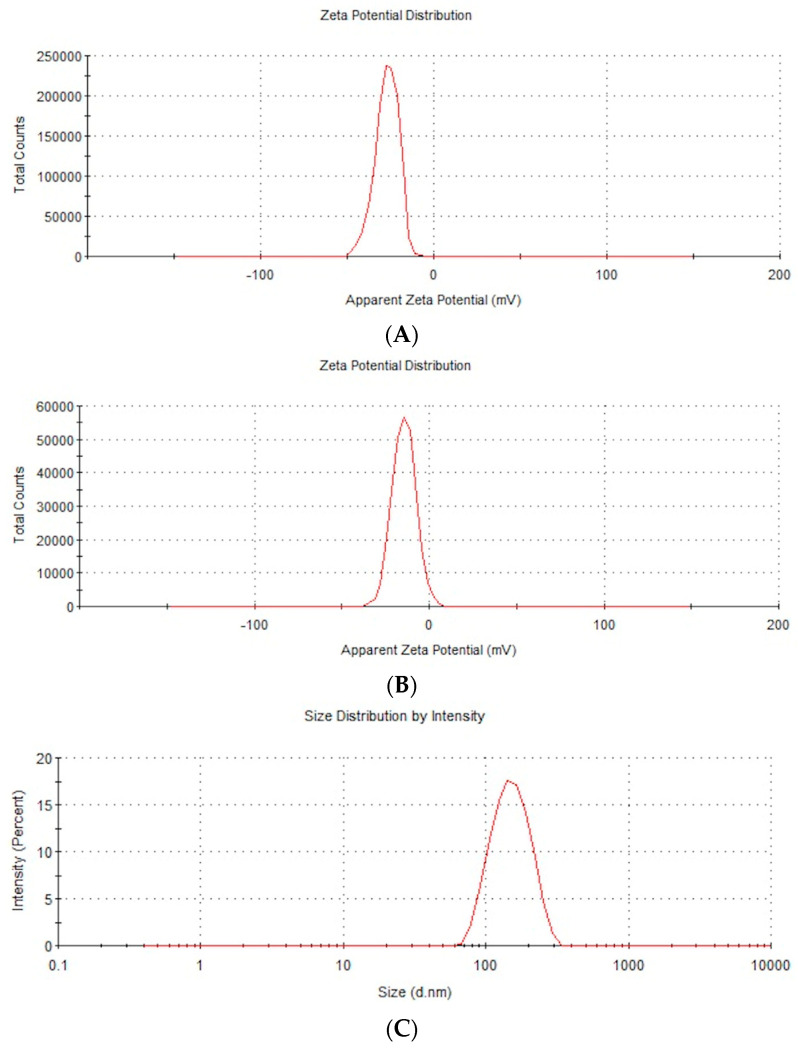

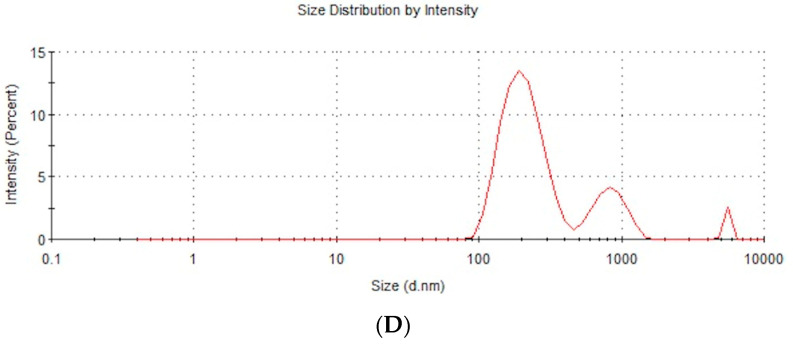
(**A**) Representative distribution of the AuNPs’ zeta potential at a concentration of 50 mg/L. (**B**) Representative distribution of the NP-FeCs’ zeta potential at a concentration of 50 mg/L. (**C**) Representative distribution of the AuNPs’ size at a concentration of 50 mg/L. (**D**) Representative distribution of the NP-FeCs’ size at a concentration of 50 mg/L.

Dynamic laser scattering under electrophoretic conditions was used to measure the zeta potential, and the Smoluchowski correction was used to calculate the zeta potential. Each sample was measured after 120 s of stabilization at 25 °C in triplicate. Each replication contained at least 20 averaged analyses. Representative repetitions of the zeta potential distribution and mobility are presented in Figure 1A–D.

The size and structure distribution of the nanoparticles was determined by analyzing transmission electron microscopy images and hydrodynamic size measurements. The NPs were observed using a JEM-2000EX (JEOL, Tokyo, Japan) transmission electron microscope. The NPs, in hydrocolloid form, were sonicated for 60 min, then applied to the forming mesh, and left to dry for 24 h. Images of the AuNPs and NP-FeCs were taken at a voltage of 200 kV. In order to analyze the hydrodynamic size of the NPs, they were sonicated for 30 min and then measured using a Zetasizer Nano ZS (ZEN3500; Malvern Instruments, Malvern, UK). The hydrodynamic size was determined using dynamic laser light scattering (DLS). Each sample was measured after 120 s of stabilization at 25 °C in triplicate. Each replication contained at least 10 averaged analyses.

### 2.3. The In Vitro Culture of BME-UV1 Cells

Bovine mammary gland cells of the BME-UV1 line used in the experiment were provided by M. Gajewska (Veterinary Medicine Faculty, Warsaw University of Life Sciences). The BME-UV1 cells were kept in appropriate DMEM/F12 medium (Thermo Fisher Scientific, Waltham, MA, USA) supplemented with 10% bovine serum and with antibiotic and antimycotic agents added (Gibco™ Antibiotic-Antimycotic, Thermo Fisher Scientific, Waltham, MA, USA). The cells were cultured in vitro in bottles for adherent cell culture at a temperature of 37 °C in an atmosphere with 5% CO_2_ in a NuAire DH AutoFlow CO_2_ incubator (Plymouth, MN, USA).

### 2.4. The Viability of BME-UV1 Cells after Incubation with AuNPs and NP-FeCs

AuNP and NP-FeC solutions were prepared according to the methods described in this paper in Section 2.1.

The viability of the BME-UV1 cells was calculated using the PrestoBlue test (Thermo Fisher Scientific, USA). The PrestoBlue test’s reagent is a ready-to-use resazurin-based solution for which cells are permeable. It can, therefore, be useful as a cell viability indicator using the reducing power of living cells. The reagent is modified by reducing the environment of viable cells and turns from blue to purple or pink in color, becoming highly fluorescent. Due to this phenomenon, the color changes can be evaluated using absorbance or fluorescence values.

BME-UV1 cells were placed in sterile 96-well plates with covers at 5 × 10^3^ cells per well. The cells were incubated for 24 h. Next, the medium was removed and 90 μL of AuNP and NP-FeC hydrocolloids at a concentration of 0.5, 1, 2.5, or 5 mg/L were added to each well. The control group consisted of cells kept in the medium without the addition of NPs. After 24 h of incubation, 10 μL of PrestoBlue reagent was added to each well and the prepared plates were incubated for 2 h at 37 °C. The absorbance value was measured at a wavelength of 570 nm in an Infinite M200 immunoenzymatic reader (Tecan, Durham, NC, USA).

### 2.5. The In Vitro Membrane Integrity of BME-UV1 Cells after Incubation with AuNPs and NP-FeCs

The amount of lactate dehydrogenase (LDH) was verified in order to evaluate the membrane integrity of the BME-UV1 cells. The assay can be used to quantitatively measure the extracellular LDH release in culture media, due to a specific enzymatic reaction. The result of the process is a red formazan product that can be spectrophotometrically measured.

Cells of the BME-UV1 line were placed in 96-well plates at 5 × 10^3^ cells per well. The plates were incubated for 24 h. Following this, the medium was removed and 90 μL of AuNP and NP-FeC hydrocolloids at a concentration of 0.5, 1, 2.5, or 5 mg/L were added to each well. The control group consisted of cells placed in the medium without the addition of NPs. After 24 h of incubation, the plates were centrifuged and 50 μL of the medium was placed in new 96-well plates. An LDH working solution was then added to each well according to the producer’s instructions (LDH Cytotoxicity Assay Kit, Thermo Fisher Scientific). The plates were incubated for 20 min at room temperature without light. In the next step, the reaction was completed by adding 50 μL of a stop solution. Absorbance values were measured at a wavelength of 490 nm in an Infinite M200 immunoenzymatic reader (Tecan, Durham, NC, USA).

### 2.6. The In Vitro Estimation of the Number of BME-UV1 Cells after Incubation with AuNPs and NP-FeCs

In order to assess the number of BME-UV1 cells, the amount of lactate dehydrogenase (LDH) released from all cells after they were lysed was evaluated. BME-UV1 cells were placed in 96-well plates at 5 × 10^3^ cells per well. The plates were incubated for 24 h. After this, the medium was removed and 90 μL of AuNP and NP-FeC hydrocolloids at a concentration of 0.5, 1, 2.5, or 5 mg/L were added per well. The control group consisted of cells placed in the medium without the addition of NPs. After 24 h of incubation, the plates were centrifuged and 50 μL of the medium was placed in new 96-well plates. An LDH working solution was then added to each well according to the producer’s instructions (LDH Cytotoxicity Assay Kit, Thermo Fisher Scientific). The plates were incubated for 20 min at room temperature without light. In the next step, the reaction was completed by adding 50 μL of a stop solution. Absorbance values were measured at a wavelength of 490 nm in an Infinite M200 immunoenzymatic reader (Tecan, Durham, NC, USA) and normalized to a measurement of 680 nm.

### 2.7. Statistical Analysis

The obtained results were analyzed using one-way analysis of variance (ANOVA) in the Prism 8 program (GraphPad Software v. 4.0, La Jolla, CA, USA). Differences between groups were estimated using Tukey’s test. The results of the survey were presented as average values with standard deviations. Differences at *p* ≤ 0.05 were considered to be statistically significant.

### 2.8. In Vitro Bacterial and Fungi Cultures

Several species of bacteria from different groups were obtained from LGC Standards (Łomianki, Poland). The examined pathogens were as follows: Gram-positive bacteria (*Staphylococcus aureus*, *Streptococcus agalactiae*, *Streptococcus uberis*), Gram-negative bacteria (*Enterococcus faecalis*, *Escherichia coli*, *Enterobacter cloacae*, *Pseudomonas aeruginosa*), and the yeast *Candida albicans*. The cultured microorganisms were kept in 20% glycerol solution under temperature conditions of −20 °C.

The pathogens were thawed and rinsed with sterile distilled water in order to remove the glycerol. In the next step, bacterial cells or fungi were added to nutrient broth medium (Bio-Rad, Warsaw, Poland) that had been sterilized in glass flasks in a Classic 2100 autoclave (Prestige Medical, Chesterfield, UK). The flasks were placed in a rotating incubator at a temperature of 37 °C (Stuart SI500 Vernon Hills, IL, USA).

### 2.9. Preliminary NP Concentrations

Bacterial cells pipetted from the cell culture and incubated for one night (at 37 °C) were used in the experiments. The experimental groups contained nutrient broth and AgNPs, CuNPs, or AgCuNPs at a concentration of 0.5, 1, or 2.5 µg/mL. The control group contained nutrient broth without the addition of NPs. Each group was prepared in three repetitions. In the next step, 100 μL of the microorganism species (at a concentration of 1 × 10^6^ microorganisms per mL) mentioned earlier were added to prepared flasks. The samples were incubated for 24 h in a rotating incubator (SI500) at a temperature of 37 °C and a rotation speed of 70 revolutions per minute. The microorganisms’ viability was calculated using the PrestoBlue test. After incubation, 90 µL of the medium was placed in 96-well plates and 10 µL of PrestoBlue reagent (Thermo Fisher Scientific) was added to each well. Each sample was placed in the well in six repetitions. The plates were incubated for 20 min at 37 °C. Absorbance was measured at a wavelength of 570 nm in an Infinite M200 immunoenzymatic reader (Tecan). The viability of the pathogens was presented as a percentage of the viability of the control group, according to the following equation:

$$\begin{array}{l} \text{X = (optical sample density × 100\%)/optical control group density}\\ \text{X = pathogen viability} \end{array}$$

### 2.10. Preparation of Mixture and Wax with the Addition of NPs

The fluid mixture consisted of 10% vegetable glycerin and 10% propylene glycol in distilled water with the addition of NPs at selected concentrations. The mixture included homogenous NPs or an AgCuAuNP complex prepared according to the description presented in Section 2.1. The NP hydrocolloids consisting of homogenous NPs or the AgCuAuNP complex were mixed with vegetable glycerin and propylene glycol in appropriate proportions at 25 °C and vortexed for 60 s.

The beeswax and olive oil mixture was heated at 37 °C to a uniform consistency. The NP hydrocolloids consisting of homogenous NPs or the AgCuAuNP complex were mixed with the prepared wax and vortexed for 5 min at 37 °C.

## 3. Results

**Figure 2:**
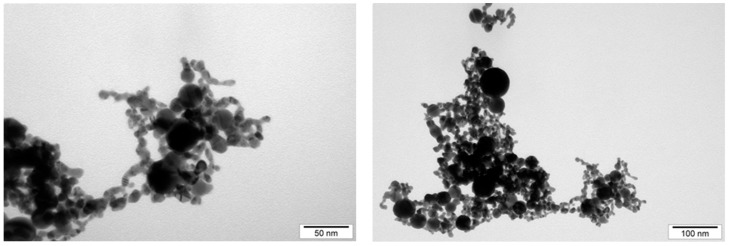
The microphotographs of the AuNPs obtained using a transmission electron microscope.

**Figure 3:**
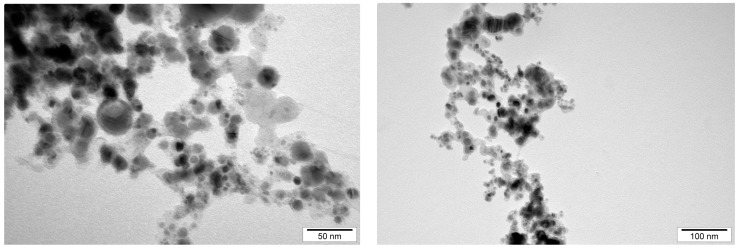
The microphotographs of the NP-FeCs obtained using a transmission electron microscope.

The size distribution was determined by analyzing images of AuNPs and NP-FeCs obtained using a transmission electron microscope, as was the hydrodynamic size (average size of agglomerates). Measurements of the hydrodynamic size were carried out using hydrocolloids at a concentration of 50 mg/L. The results of the hydrodynamic size measurements and the sizes of the nanoparticles measured using transmission electron microscopy images are shown in Table 1. In addition, the structure of the AuNPs and NP-FeCs was determined based on images obtained using electron microscopy. Selected microphotographs are shown in Figure 2 and Figure 3.

### 3.1. The Estimated Viability of the BME-UV1 Cells after Incubation with AuNPs and NP-FeCs

**Figure 4:**
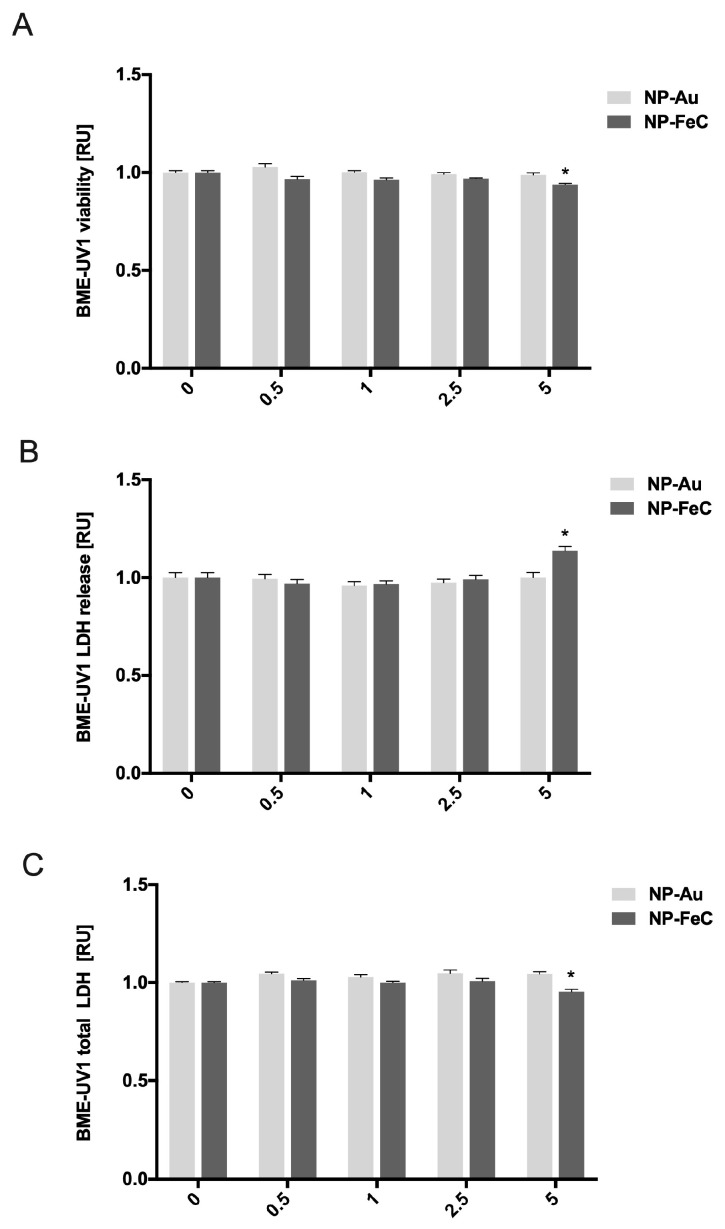
The viability of BME-UV1 cells estimated using a PrestoBlue assay (**A**). Evaluation of BME-UV1 cell membrane integrity (**B**). Determination of the total number of BME-UV1 cells using the LDH assay (**C**). * Statistically significant differences at *p* < 0.05.

The in vitro viability of the BME-UV1 cells was determined using a PrestoBlue assay, measuring the redox activity of the cells. AuNPs at concentrations of 0.5, 1, 2.5, and 5 mg/L had no cytotoxic effect on the epithelial mammary cells (BME-UV1). The viability of the selected cell line decreased only when cells were treated with NP-FeCs at the highest concentration (5 mg/L). The results for cell viability after 24 h of incubation with AuNP and NP-FeC hydrocolloids are shown in Figure 4A. NP-FeCs at concentrations of 0.5, 1, and 2.5 mg/L had no cytotoxic effect on the epithelial mammary cells (BME-UV1).

The integrity of the BME-UV1 cells’ membranes was assessed using the LDH Cytotoxicity Assay Kit (Thermo Fisher Scientific, USA). The cell membrane’s integrity was compromised after treatment with NP-FeCs at a concentration of 5 mg/L. The results for the cell membrane’s integrity after 24 h of incubation with AuNP and NP-FeC hydrocolloids are shown in Figure 4B.

The total number of BME-UV1 cells was assessed using the LDH Cytotoxicity Assay Kit (Thermo Fisher Scientific, USA) by determining the total amount of LDH. The number of cells changed after being treated with NP-FeCs at a concentration of 5 mg/L. The results for the cell counts after 24 h of incubation with AuNP and NP-FeC hydrocolloids are shown in Figure 4C.

### 3.2. The Cytotoxic Effect of AgNPs, CuNPs, AuNPs, and NP-FeCs on Pathogen Viability

The obtained results suggested that metal NPs can decrease the viability of the selected mastitis bacteria, *Staphylococcus aureus*, *Streptococcus agalactiae*, *Streptococcus uberis*, *Enterococcus faecalis*, *Escherichia coli*, *Enterobacter cloacae*, and *Pseudomonas aeruginosa*, and the yeast *Candida albicans* even at low concentrations (Table 2). The results included the average viability for each group and the standard error (SE) after incubation with homogenous NP hydrocolloids at various concentrations (0.5–5 ppm). The presented data were statistically significant for all the examined groups (*p* < 0.01).

The viability of the pathogens used in the experiment decreased in three of the experimental groups: AgNPs, CuNPs, and AuNPs.

The strongest antimicrobial properties—decreasing the viability of *S. uberis*, *S. agalactiae*, and *E. faecalis* by 50% or more—were observed for AgNPs at a concentration of 5 ppm. The results for *E. cloacae* pointed to the strongest biocidal effect having occurred in the AuNPs5 group (AuNPs at a concentration of 5 ppm; see Table 2). Similar results were observed in the CuNPs5 group for *S. uberis* and *E. faecalis*, where the viability of the pathogens decreased by 45%.

Conversely, the group with NP-FeCs added promoted bacterial growth, which was indicated by viability values >100% (the control group’s viability was always 100%). The exception was the *S. aureus* group, where the viability varied from 96.05% to 99.97%. Therefore, NP-FeCs were not included in later parts of the experiment because of their lack of antimicrobial properties.

### 3.3. The Cytotoxic Effect of the AgCuAuNP Complex on Pathogen Viability

The cytotoxic effect of the AgCuAuNP complex was observed during the experiment (Table 3). The combination of the three different metal NP types produced lower viabilities at all concentrations for all tested pathogens. The NPs’ strongest biocidal effect was observed at the highest concentration (5 ppm). The difference between the 2 ppm and 5 ppm concentrations was relatively small in the case of the selected pathogen species. The presented data were statistically significant for all the examined groups (*p* < 0.01).

### 3.4. The In Vitro Antimicrobial Properties and Cytotoxic Effect of the AgCuAuNP Complex

The wax that included homogenous NPs had lower antimicrobial properties, decreasing the pathogens’ viability by 14–20% (Table 4). The exception was the wax with homogenous NPs applied to *S. agalactiae*, which decreased the viability by 47% in the case of AgNPs and AuNPs. The AgCuAuNP complex had the weakest antibacterial activity against *S. aureus* (71%). The viability of *E. coli*, *S. agalactiae*, and *C. albicans* was 55%, 57%, and 66%, respectively.

The results of adding homogenous NPs to the liquid mixture were similar to those for the wax groups. Similar data were obtained for the AgCuAuNP complex, with the strongest antimicrobial activity being observed in the *E. coli* group. The obtained results were statistically significant for all groups (*p* < 0.01).

## 4. Discussion

### 4.1. Nanotechnology and NPs’ Properties

Inflammation of the tissue of the bovine mammary gland is still the most common disease found in high-yielding dairy cattle. It causes significant deterioration of the cows’ welfare and affects the parameters and cytological quality of milk [11].

The antimicrobial properties of metal NPs are associated with various factors, including their stability in hydrocolloid form, which results in no agglomeration phenomenon and stronger biocidal properties because the NPs’ surfaces are not limited [12]. In this study, AuNPs and NP-FeCs (Table 1) had zeta potential values of −28.4 mV and −18.5 mV, respectively. These results suggest weak antibacterial properties, because the limit of NP stability is ±30 mV. Hence, the average size of the agglomerates was higher for NP-FeCs than for AuNPs (342.9 nm vs. 148.3 nm, respectively). The physicochemical properties of AgNPs, AuNPs, and CuNPs, obtained from commercial sources have been confirmed in previous studies [13,14,15].

### 4.2. The Influence of NPs on the Viability of Bovine Mammary Gland Cells

The toxic effect of NPs on water organisms has been observed in several papers [16,17,18]. The exposure of water organisms to NPs usually causes several problems, for example, effects on larval development, impaired reproduction, or increased oxidative stress [17,18]. Moreover, for aquatic animals, AgNPs are often considered to be more toxic than CuNPs [16]. At the same time, NPs are used in veterinary and human medicine, for example, in cancer treatment or disinfectant products. An interesting phenomenon was described by Matuszewski et al. [19], proving that in ovo inoculation with calcium carbonate NPs positively influences bone mineralization if the process takes place during chicken embryogenesis [19]. The described correlations have not been completely examined, and further studies—both in vitro and in vivo—are necessary to evaluate the long-term effects of NPs on animals and humans.

Studies focusing on the long-term effects of NPs on bovine cells are surprisingly limited [13,20]. The safety of animals and humans is a crucial issue if NPs are to be used as an alternative antimicrobial agent in the future. According to Jagielski et al. [20], AgNPs have no cytotoxic effect on mammary gland cells if used at concentrations of up to 1–4 mg/L [20]. Similar results were obtained by Kalińska et al. [13]. However, study results vary among scientists and some papers point out that NPs can be toxic for humans [21]. Nevertheless, available references are still limited in the case of the BME-UV1 cell line. Kalińska et al. [13] revealed that AgNP, AgCuNP, and CuNP concentrations of 0.5–2.5 mg/L increase the likelihood of spontaneous leaking of LDH by 20–60% (*p* < 0.05). The authors of this paper did not observe an increase in LDH release in the case of AuNPs and NP-FeCs, and no negative impact on BME-UV1 cells was observed. The use of an LDH assay can be a valuable factor in assessing the potential toxicity of new antimicrobial agents, as cellular proteins can adhere to NPs as a protein corona. But the LDH assay should always be carefully included for evaluating the NPs’ effect on cell viability [22].

### 4.3. The Antimicrobial Properties of NPs

The increasing number of strains that are resistant to antibiotics is a clearly observed phenomenon [7,23]. Scientists are therefore struggling to invent new agents that could be used as alternatives to drug treatments [2]. Another important issue in current studies is the development of rapid diagnostic methods for mastitis that are accurate regarding at least the type of pathogen and preferably the species, for example, *Staphylococcus aureus*. This would contribute significantly to speeding up the time for diagnosis and the implementation of appropriate treatment. For this reason, markers for udder inflammation in cows are still being investigated. Some studies have indicated that whey proteins may act as markers [24], but there are no clear bovine markers that are currently considered to be indicators of developing inflammation in the udder. Somatic cell count (SCC) growth is still the most commonly used symptom for the diagnosis of subclinical inflammation. Some of the most promising solutions for prophylaxis of inflammation are plant extracts, fungi extracts, phage therapy, lysozyme, lactoferrin, bacteriocins, and NPs [13,24,25,26,27,28].

Previous studies have revealed that NPs can be used in cases of several bovine diseases, for example, mastitis [13] and lameness [14]. This solution is also promising due to the fact that NPs do not negatively influence bovine mammary tissue [14]. Another important issue is whether it is possible to obtain high-quality NPs from commercial sources [13,14], which is an important factor in planning the further development of NP products. Studies using NPs in pre-milking and post-milking disinfectant products are limited, but in vitro experiments suggest that AgNPs and CuNPs can reduce pathogen viability by 50% [16]. The antimicrobial properties of some metal NPs are well known, for example, AgNPs and CuNPs [13,14,15,26]; the idea of using the synergistic effect of at least two different NPs has also previously been presented [13,26]. In the case of mastitis, AuNPs, PtNPs, and NP-FeCs demonstrate weaker antimicrobial properties even at high concentrations [14,29], and these results are similar to the data presented in this paper.

### 4.4. Teat Sealant in Dry Cow Therapy

The authors of this study estimate the average cost of mastitis incidences to be a minimum of €240/cow/year [30]. Similar data from other European countries estimate that the total mastitis cost per cow per year ranges from €261 to €483 [31,32]. In contrast, estimated average losses due to mastitis in the U.S. are at a minimal level of $131/cow/year [33].

Dry cow therapy is a crucial point in the production cycle. In accordance with EU Regulation 2019/6 on veterinary medicinal products that repealed Directive 2001/82/EC of 28 January 2022, regulations force veterinarians to reduce the use of antibiotics in animal production and are banning the use of some antibiotics as a method of prophylaxis. The amount of antibiotics used in the EU is highly disturbing, with an average of 84.4 mg/PCU (standardization unit), with some countries having almost double this value, for example, Cyprus, Poland, Hungary, Italy, Spain, and Portugal [34]. In addition, the guidelines in the Green Deal Industrial Plan indicate the need to reduce antibiotic use by 50% as early as 2030 [34,35].

In conclusion, the use of antibiotics as the most common form of mastitis treatment in dairy cows also requires a change in approach to reduce antibiotic use. One of the most popular procedures is combining antibiotic treatment with teat sealants during dry cow therapy. The first teat sealants were created in the early 1970s and were based on bismuth subnitrate; it is still the most common substance used. There are several papers proving that the use of teat sealants on dry cows, in addition to antibiotic treatment, has a positive influence, resulting in lower SCCs at the beginning of lactation and even higher milk production [36,37,38,39].

Based on the results obtained from the in vitro testing of wax plugs and teat disinfectant liquid, a decrease can be observed in the viability of the pathogens that most often cause mastitis in cows, resulting in a decrease in SCCs in milk and an increase in the milk yield of cows. ElAshmawy et al. [40] showed that there was an increase in milk production (by 1.84 kg/day) in cows treated with antibiotics and intramammary sealants compared to the control group. The authors also demonstrated a reduction in the SCCs during the first 150 days of lactation in cows treated with antibiotics and sealants and with sealants alone compared to controls. Molina et al. [37], in their study, used a combination of the antibiotic cloxacillin and an injectable sealant containing bismuth nitrite in its formulation. The authors showed that there was a decrease in the incidence of new mastitis infections by 8% in the early postpartum period after using the antibiotic and sealant compared to controls using the antibiotic alone. It was also observed that there was a reduction in the amount of *E. coli* bacteria in the milk of cows after calving compared to the pre-delivery sample after using the sealant. In contrast, the use of injectable sealants had no effect on the frequency of streptococcal isolation. Clabby et al. [41] carried out an experiment in which they dried one experimental group of cows with dystrophin sealants and an antibiotic and the other with the dystrophin sealant alone. The authors found that cows dried with the teat sealant alone had higher SCCs and were more likely to develop udder infections during the next lactation compared to cows dried with both the antibiotic and the sealant.

In conclusion, it is likely that NPs have high potential to be biocidal agents in future mastitis treatment and prevention also in dried cows. However, the obtained in vitro results require further studies using strains isolated from cows with clinical mastitis. Thus, the next step should be experiments with pathogens isolated from cows with mastitis, since these strains (including *S. aureus* or *E. coli*) may have different resistance to the tested NPs. For this reason, it is necessary to verify their biocidal properties also against field strains.

## 5. Conclusions

Limiting the phenomenon of antibiotic resistance is mainly related to discontinuing the use of antibiotics as a prophylaxis method. Before beginning treatment, it is necessary to identify the etiological factor so the appropriate drug can be selected in the appropriate dosage. Sales of antibiotics in some countries continue to increase, especially in the livestock sector, which poses a serious threat to the health of consumers. In order to prevent the increasing problem of antibiotic resistance in animal livestock, it is necessary to look for alternative agents and forms of treatment that will significantly reduce the use of antibiotics in the future. The conducted in vitro studies indicate that the use of AuNPs, AgNPs, CuNPs, and their triple complexes reduces the viability of the most common mastitis pathogens, i.e., *S. aureus*, *E. coli*, and *S. uberis*. Moreover, these NPs are not toxic to bovine mammary gland tissue. This suggests that in the future, NPs could be a new alternative in the prevention and treatment of mastitis in dry cows, but further in vivo studies are needed.

## Figures and Tables

**Table 1:** Size distribution and structure of AuNPs and NP-FeCs.

	Zeta Potential	Hydrodynamic Size	NP Diameter	NP Structure
	(mV)	(nm)	(nm)	
Measurement method	Mobility	Dynamic light scattering (DLS)	Transmission electron microscopy	Transmission electron microscopy
Defined parameter	Zeta potential	Average size of agglomerate	Size of single NPs	NP form
AuNPs	−28.4	148.3	15–75	Spherical
NP-FeCs	−18.5	342.9	10–80	Spherical

**Table 2:** The in vitro viability of pathogens used in the experiment after incubation with homogenous NP hydrocolloids at various concentrations (0.5–5 ppm) presented as a percentage of the control group (%).

	*Staphylococcus aureus*		*Escherichia coli*		*Streptococcus agalactiae*		*Streptococcus uberis*		*Enterococcus faecalis*		*Enterobacter cloacae*		*Pseudomonas aeruginosa*		*Candida albicans*	
	Average	SE	Average	SE	Average	SE	Average	SE	Average	SE	Average	SE	Average	SE	Average	SE
Control group	100.00	0.013	100.00	0.050	100.00	0.076	100.00	0.036	100.00	0.027	100.00	0.038	100.00	0.029	100.00	0.022
AgNPs0.5	79.32	0.047	72.20	0.020	80.53	0.009	63.94	0.035	91.66	0.041	80.07	0.043	84.99	0.037	88.97	0.026
AgNPs1	77.54	0.056	75.43	0.039	76.47	0.025	58.36	0.042	72.78	0.019	63.38	0.053	82.05	0.062	78.20	0.030
AgNPs2	74.87	0.034	58.46	0.031	64.64	0.026	56.50	0.025	69.31	0.067	55.45	0.013	81.37	0.013	77.42	0.033
AgNPs5	63.91	0.069	64.76	0.046	48.42	0.030	49.44	0.015	52.24	0.061	64.35	0.020	73.59	0.040	73.00	0.039
AuNPs0.5	97.08	0.026	91.04	0.076	97.43	0.026	91.11	0.035	93.72	0.044	84.11	0.024	87.98	0.035	98.10	0.047
AuNPs1	87.98	0.129	86.14	0.036	90.44	0.023	86.84	0.018	82.61	0.005	67.15	0.041	86.25	0.029	94.37	0.011
AuNPs2	84.24	0.081	88.57	0.008	88.59	0.058	84.40	0.017	80.66	0.021	64.10	0.026	84.10	0.011	86.62	0.038
AuNPs5	76.27	0.052	87.46	0.036	82.84	0.011	78.57	0.042	60.05	0.026	46.89	0.003	60.43	0.037	76.82	0.034
CuNPs0.5	92.64	0.055	85.94	0.032	95.51	0.023	89.47	0.038	86.74	0.036	85.50	0.025	90.80	0.020	97.35	0.036
CuNPs1	88.75	0.089	83.08	0.034	91.73	0.012	73.90	0.015	80.11	0.017	72.54	0.030	79.40	0.018	91.04	0.029
CuNPs2	81.05	0.144	78.75	0.028	85.67	0.037	70.97	0.031	67.30	0.018	60.36	0.042	70.81	0.019	86.17	0.033
CuNPs5	84.07	0.099	71.05	0.004	79.60	0.036	54.41	0.048	55.86	0.021	70.35	0.029	67.11	0.031	88.28	0.036
NP-FeC0.5	96.05	0.044	101.02	0.030	130.37	0.069	134.41	0.154	104.20	0.025	125.42	0.066	99.58	0.050	138.43	0.054
NP-FeC 1	99.97	0.052	110.80	0.052	137.72	0.112	159.15	0.050	159.60	0.033	125.60	0.043	104.35	0.030	123.08	0.018
NP-FeC 2	96.91	0.112	100.12	0.047	128.33	0.081	132.87	0.041	144.87	0.033	129.78	0.050	108.90	0.070	130.89	0.089
NP-FeC 5	98.69	0.170	97.82	0.016	125.65	0.019	139.84	0.082	146.80	0.062	132.55	0.054	122.71	0.053	135.51	0.040
	*p* < 0.05		*p* < 0.01		*p* < 0.01		*p* < 0.01		*p* < 0.01		*p* < 0.01		*p* < 0.01		*p* < 0.01	

**Table 3:** The in vitro viability of pathogens used in the experiment after incubation with NP complexes at various concentrations (0.5–5 ppm) presented as a percentage of the control group (%).

	*Staphylococcus aureus*		*Escherichia coli*		*Streptococcus agalactiae*		*Streptococcus uberis*		*Enterococcus faecalis*		*Enterobacter cloacae*		*Pseudomonas aeruginosa*		*Candida albicans*	
	Average	SE	Average	SE	Average	SE	Average	SE	Average	SE	Average	SE	Average	SE	Average	SE
Control group	100.00	0.021	100.00	0.077	100.00	0.022	100.00	0.062	100.00	0.043	100.00	0.028	100.00	0.035	100.00	0.041
AgAuCuNPs0.5	80.40	0.036	76.21	0.109	79.76	0.021	80.09	0.025	87.70	0.029	91.28	0.045	81.79	0.031	85.01	0.041
AgAuCuNPs1	72.21	0.031	57.11	0.021	60.75	0.020	65.53	0.058	57.75	0.030	73.03	0.018	74.38	0.028	86.22	0.061
AgAuCuNPs2	70.88	0.033	61.91	0.053	58.77	0.026	55.54	0.014	58.99	0.042	78.35	0.054	77.40	0.025	82.18	0.013
AgAuCuNPs5	70.38	0.016	57.99	0.051	56.67	0.032	53.99	0.022	60.03	0.018	80.37	0.044	75.21	0.035	75.45	0.010
	*p* < 0.01		*p* < 0.01		*p* < 0.01		*p* < 0.01		*p* < 0.01		*p* < 0.01		*p* < 0.01		*p* < 0.05	

**Table 4:** The in vitro viability of pathogens used in the experiment after incubation with NP complexes and the liquid mixture (mix), and NP complexes and wax (wax) at a concentration of 1 ppm. The obtained results are presented as a percentage of the control group (%).

	*Staphylococcus aureus*		*Escherichia coli*		*Streptococcus agalactiae*		*Candida albicans*	
	Average	SE	Average	SE	Average	SE	Average	SE
Control group	100.00	0.15	100.00	0.11	100.00	0.07	100.00	0.03
Wax	91.81	0.07	84.94	0.08	79.76	0.07	92.68	0.06
AgNPs1+ wax	85.10	0.19	78.65	0.04	53.73	0.02	73.22	0.03
AuNPs1+ wax	92.14	0.05	84.11	0.04	53.73	0.02	77.67	0.03
CuNPs1+ wax	86.70	0.04	81.12	0.09	58.43	0.02	86.90	0.03
AgAuCuNPs1+ wax	70.78	0.03	55.09	0.03	57.74	0.04	66.30	0.04
Mix	85.67	0.09	90.22	0.06	90.14	0.04	96.76	0.05
AgNPs1+ mix	79.83	0.02	75.30	0.07	57.81	0.02	77.27	0.04
AuNPs1+ mix	84.31	0.06	84.11	0.10	58.34	0.03	85.89	0.05
CuNPs1+ mix	85.29	0.08	81.12	0.12	82.08	0.04	83.26	0.03
AgAuCuNPs1+ mix	77.76	0.11	57.38	0.04	62.66	0.08	64.44	0.03
	*p* < 0.01		*p* < 0.01		*p* < 0.01		*p* < 0.01	

## Data Availability

Data are available on request.
